# Channelopathy pathogenesis in autism spectrum disorders

**DOI:** 10.3389/fgene.2013.00222

**Published:** 2013-11-05

**Authors:** Galina Schmunk, J. Jay Gargus

**Affiliations:** ^1^Department of Physiology and Biophysics, University of CaliforniaIrvine, CA, USA; ^2^UCI Center for Autism Research and Treatment, School of Medicine, University of CaliforniaIrvine, CA, USA; ^3^Department of Pediatrics, Section of Human Genetics, University of CaliforniaIrvine, CA, USA

**Keywords:** calcium, mTOR, Fragile X syndrome, tuberous sclerosis, Rett syndrome, Prader-Willi syndrome, Angelman syndrome

## Abstract

Autism spectrum disorder (ASD) is a syndrome that affects normal brain development and is characterized by impaired social interaction as well as verbal and non-verbal communication and by repetitive, stereotypic behavior. ASD is a complex disorder arising from a combination of multiple genetic and environmental factors that are independent from racial, ethnic and socioeconomical status. The high heritability of ASD suggests a strong genetic basis for the disorder. Furthermore, a mounting body of evidence implies a role of various ion channel gene defects (channelopathies) in the pathogenesis of autism. Indeed, recent genome-wide association, and whole exome- and whole-genome resequencing studies linked polymorphisms and rare variants in calcium, sodium and potassium channels and their subunits with susceptibility to ASD, much as they do with bipolar disorder, schizophrenia and other neuropsychiatric disorders. Moreover, animal models with these genetic variations recapitulate endophenotypes considered to be correlates of autistic behavior seen in patients. An ion flux across the membrane regulates a variety of cell functions, from generation of action potentials to gene expression and cell morphology, thus it is not surprising that channelopathies have profound effects on brain functions. In the present work, we summarize existing evidence for the role of ion channel gene defects in the pathogenesis of autism with a focus on calcium signaling and its downstream effects.

## AUTISM AND AUTISM SPECTRUM DISORDERS

Autism is a disease that dramatically affects brain function early in development. Its societal consequence and costs are enormous, currently costing over $130 billion per year in the USA alone (Mandell and Knapp, 2012). Worse, its prevalence has been increasing over the last decade with current Center for Disease Control estimates suggesting that nearly 2% of children are affected (Blumberg et al., 2013).

Symptoms of autism typically start between the second and third year of life and cause problems of a wide range of severity in various areas of development. It is a neurodevelopmental disorder with three core behavioral features: (1) qualitative impairment in social skills, (2) delayed or disordered language and communication skills, and (3) restricted and repetitive behaviors. The autistic spectrum disorders (ASD), the preferred term for this broad constellation of pervasive developmental disorders, all share the same three characteristic core deficits. The clinical diagnosis of autism is made by specially trained physicians and psychologists who perform evaluations focused on detailed histories and behavioral observations. ASD diagnosis for research studies is stricter, more time consuming and quantitative, but even at this most refined level ASD remains a group of developmental disorders that are only behaviorally, not yet pathophysiologically, defined (Filipek, 2005). With the May 2013 publication of the new American Psychiatric Association Diagnostic and Statistical Manual (DSM-5), all autism subtypes will be merged into one umbrella diagnosis of ASD.

Objective quantifiable biochemical markers of this disease have been very hard to come by. However, the high heritability (h^2^) of ASD, which while still controversial has been calculated at up to 90%, makes it one of the most highly heritable behavioral disorders (Hallmayer et al., 2011; Devlin and Scherer, 2012). This provides powerful assurance that genes and the biochemical pathways they subserve underlie the phenotype. Identifying such alterations in affected individuals would provide an added dimension to the phenotype, perhaps refining more coherent subgroups of ASD (endophenotypes). Environmental impacts, also clearly implicated by monozygotic twins who are discordant for ASD (Hallmayer et al., 2011), may be best understood to perturb these same pathways. Those genes additionally lead to personalized medicine, potentially serving as new molecular diagnostics of the disease and as targets for the development of new classes of highly selective medications, much as has been the case for cancer. Evidence suggests a complex multigenic etiology of many, if not most cases of ASD. Emergent evidence from the genetic architecture is beginning to implicate aberrant neuronal signaling in ASD, and several cases strongly implicate a channelopathy pathogenesis of the disorder.

## DEFECTIVE CHANNEL FUNCTION IN AUTISM

Ion channels are a large family of transmembrane proteins that provide ions a passive pathway through which they can rapidly diffuse down their electrochemical gradient across the hydrophobic barrier of the membrane (Hille, 2001). The standing electrochemical gradients that drive passive ion movements though channels are established by energy dependent active transport mechanisms such as ion pumps and ion carriers (Gargus, 2008). Ion channels conduct ions four orders of magnitude faster than pumps and carriers, so in many ways channels act like highly selective water filled pores that can be opened and closed in a controlled fashion (gated) to allow a specific ion species to flow. This causes a miniscule chemical flux but an appreciable electrical current sufficient to change the membrane potential toward the Nernst potential of the conducted ion. Since the sodium-potassium ATPase ion pump maintains a cytoplasm high in K^+^ and low in Na^+^^,^ opposite to the cell exterior, the Nernst potential is interior negative for K^+^ and interior positive for Na^+^ as it is for Ca^2^^+^. The channel’s predominant permeant ion species is dictated by the nature of the channel’s selectivity filter. At rest the predominating membrane permeability is for K^+^. This means that an interior positive depolarization is created by opening Na^+^ and Ca^2^^+^ channels, an increasingly negative hyperpolarization created by opening K^+^ channels, and, since the Cl^-^ Nernst potential usually is near the resting potential, a stabilization of the membrane potential is created by opening Cl^-^ channels.

**FIGURE 1:**
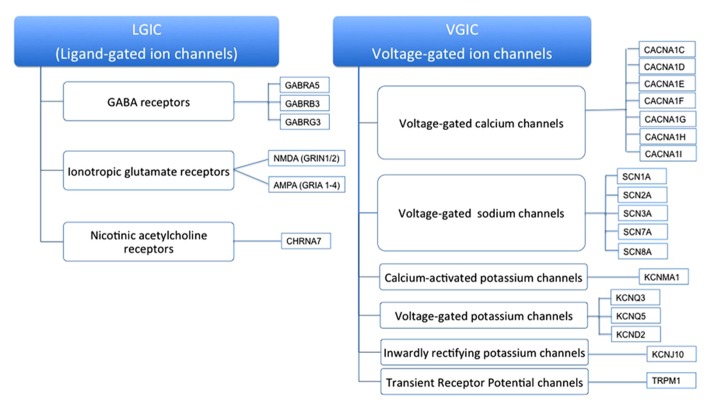
**Ion channel families and their mechanism of gating**.

Ion channel families vary in their mechanism of gating (**Figure 1**). One large family of channels gate by sensing changes in the electrical potential across the membrane – the voltage-gated ion channels. These channels respond to a membrane potential change by undergoing a conformational change from “closed” to “open.” In the “open” conducting state the channel’s own ionic current flows and thereby further alters the membrane potential. This behavior is critical to their function in perpetuating a propagating action potential (AP). As a patch of membrane begins to depolarize, voltage-gated Na^+^ and Ca^2^^+^ channels begin to open, increasing the membrane permeability to sodium and calcium, driving the membrane potential further toward *their* inside-positive Nernst potential, and hence explosively driving still more adjacent voltage-gated channels to open. Ultimately these channels intrinsically “inactivate” to cease conducting. Finally, voltage-gated K^+^ channels open to repolarize the membrane, converting the dormant “inactive” sodium and calcium channels into a “closed” (but openable) state again, in preparation for conducting another AP.

A second large class of channels play a role in initiating an AP by inducing the triggering depolarization – the ligand-gated ion channels. They gate in response to the channel protein, typically located at the synaptic junction between cells, binding a ligand released into the synapse. The binding of a wide range of extracellular and intracellular diffusable ligands is able to directly gate ion channels, and many of these ligands are classical synaptic neurotransmitters such as acetylcholine or dopamine. In addition, a large family of ion channels is indirectly gated by ligands, many by the same neurotransmitters mentioned above, but in this case neurotransmitter binding occurs to a heptahelical G-protein coupled receptor (GPCR) and the channel is activated by a second messenger ligand, such as cyclic AMP, or a covalent modification, such as protein phosphorylation.

Like sodium, calcium passively enters the cytoplasm across the plasma membrane and is cleared from the cytoplasm to a level far below extracellular levels by a host of ion pumps and carriers at the expense of metabolic energy. For most ion channels it is predominantly the electrical consequences of channel activation that underlie their physiology and pathophysiology, but calcium is an important exception to this rule, since it plays an additional critical role in coupling electrical activity to biochemical pathways. Similar to sodium, calcium is eliminated back out across the plasma membrane, but it is also uniquely sequestered for subsequent rapid release within intracellular calcium storage sites (Brini and Carafoli, 2011). Cytosolic calcium signals thus originate by either the rapid release of the intracellular stores through intracellular ion channels or by extracellular calcium entering through ion channels across the plasma membrane. The intracellular calcium release channels have complex gating that includes responsiveness to plasma membrane ion channel protein voltage-sensitive conformational changes, changes in levels of cytosolic signaling intermediates, such as inositol 1,4,5-triphosphate (IP3) and changes in cytosolic calcium levels. Until recently the endoplasmic reticulum (ER) had been thought to contain the only dynamic intracellular pool of ionized calcium to participate in cellular signaling. This intracellular store could be rapidly released via intrinsic ER channels, the inositol 1,4,5-triphosphate receptors (IP3R) and the ryanodine receptors (RyR). Once released, this calcium would activate a host of kinases, ion channels and transcription factors, and then be resequestered via the ER’s calcium ATPase pump (SERCA). While mitochondria have long been known to sequester the vast majority of intracellular calcium, only relatively recently has the dynamic nature of this mitochondrial calcium pool been recognized (Spät et al., 2008; Szabadkai and Duchen, 2008) and shown to communicate with the ER in the generation of rapid calcium signals, forming a bidirectional link between energy metabolism and cellular signals transmitted via changes in the cytosolic free calcium ion concentration (Danial et al., 2003; Patterson et al., 2004; Hayashi and Su, 2007).

Calcium signals are one of the most universal and ancient of cellular signals (Berridge et al., 2000). It is a versatile biological signal, known to regulate membrane potential, ion transporters, kinases, transcription factors and even cell morphology. It is therefore not surprising that a diverse host of diseases are coming to be recognized to be caused by disruptions of intracellular calcium homeostasis. This is an emerging pathophysiological mechanism of disease, a *calciumopathy* (Stutzmann et al., 2006; Bezprozvanny and Gargus, 2008; Betzenhauser and Marks, 2010; Feske, 2010; Cain and Snutch, 2011), a special subset of the ion channel channelopathy diseases (Gargus, 2003, 2008, 2009).

## ASD IN TIMOTHY SYNDROME HAS A CALCIUM CHANNELOPATHY PATHOGENESIS

The phenotype of Timothy syndrome (TS) involves multiple systems and specifically heart, brain, immune and skin cells. It includes mild dysmorphology of the face and syndactyly of fingers and toes, suggesting a perturbation of developmental signals. It is a simple autosomal dominant syndromic disease with high penetrance, implying that one defective copy of a single specific gene is sufficient to produce the full spectrum of disease. Clinically TS is predominated by prolonged ventricular repolarization and the lethal cardiac arrhythmia syndrome long QT (LQT), so called because of its characteristic EKG finding of a rate-corrected QT (QTc) interval of between 480 and 700 ms (Splawski et al., 2004). In addition to the dysmorphology, other variable extra-cardiac symptoms include seizures, hypotonia, immune deficiency and hypoglycemia. Remarkably, over 80% also have ASD (Splawski et al., 2004, 2005, 2011). The same rare specific allele of *CACNA1C,* a gene that encodes the “cardiac-expressed” voltage-gated calcium channel, was found to cause TS in all 12 original *de novo* unrelated cases (Splawski et al., 2004), suggesting that there must be only a very limited range of changes to channel function that create the diverse tissue phenotypes.

Long QT helped establish our understanding of channelopathy pathogenesis and it has now been shown to be caused by mutations in all of the cardiac ion channels that contribute to the ventricular AP (Bokil et al., 2010). The pathogenic alleles in these eight ion channel loci and four loci encoding channel-interacting proteins (*LQT 1–12*) all prolong the repolarization of the working myocardium, prolonging the QT interval and setting the stage for a fatal arrhythmia. Like most of the other LQT mutations, TS (also called LQT8) is a simple monogenic dominant channelopathy, but unlike the others is also highly penetrant for a neurodevelopmental phenotype on the autism spectrum (Splawski et al., 2004, 2005, 2011).

Since so much is understood about the pathogenesis of LQT and the biophysics of the ion channels involved, and since TS makes it so clear that a specific mutation in this calcium channel causes both LQT *and *autism, TS holds an incomparable potential to reveal the pathophysiology of autism. The TS mutant channel expressed in the heart is also expressed in the neurons of the brain, and it must cause the symptoms in both organs since TS is a simple monogenic disease causing both phenotypes.

Only rare missense alleles have been recognized at the *CACNA1C* locus, and the specific recurrent *de novo* TS mutation, G406R, is located in the minor alternatively spliced exon 8A of the gene. Two other alleles in this locus cause a very similar syndrome, but without the syndactyly. These are found in exon 8, not 8A, suggesting cutaneous expression of only the minor transcript (Splawski et al., 2005). The two exons are mutually exclusive, with the vast majority of the mRNA containing exon 8, and both exons encoding the same protein domain. The splicing is developmentally regulated and is mediated by the polypyrimidine tract binding protein PTB (Tang et al., 2011). One of the exon 8 alleles produces exactly the same G406R missense as the classic TS mutation, but causes a severe early lethal disease, likely because of the higher abundance of this transcript isoform. The other allele in this exon, G402S, was only found in a mosaic individual, suggesting that most mutations in this gene are not compatible with viability. Indeed, even for the classical TS alleles somatic mosaicism seems to play a significant role in the variability of this syndrome (Etheridge et al., 2011). More recently, a novel *de novo* TS allele was identified in constitutively expressed exon 38, A1473G (Gillis et al., 2012). This caused the full TS syndrome, including the minor transcript phenotype of syndactyly, but it was severe like other major transcript alleles, and also caused stroke. The position of this mutation in the channel protein is very similar to the position of the G402S mutation, only in a different “pseudo-monomer” domain of the pseudo-tetrameric structure of this large channel protein. It suggests a special function for the end of transmembrane segment 6, since this novel lesion is three amino acids away from the end of segment 6 in Domain IV whereas G402S is in the same position in Domain I and G406R is nearby. A conserved structural motif containing these mutated amino acids is found in all four pseudo-monomer domains and they appear to tightly interact with one another to form the closed state of the channel pore (Depil et al., 2011). There are additional suggestions that this domain plays a role in the oligomerization of these channels into synchronized channel clusters capable of enhanced calcium signaling (Dixon et al., 2012), potentially through interaction with anchoring proteins, such as AKAP150 (Cheng et al., 2011) since a TS mutation alters this molecular function as well. Although highly suggestive clinical findings have been observed with the A1473G mutant allele, functional studies of this novel allele have not yet been performed (Gillis et al., 2012).

The TS channel conducts a major component of the inward calcium current underlying the depolarized QT interval. A lengthening of the QT to produce the LQT characteristic of the syndrome suggests that excess current is conducted by the mutant channel. This is supported by the finding that the two missense alleles at this locus that cause the short QT Brugada syndrome, A39V and G490R, are loss-of-function lesions (Antzelevitch et al., 2007; Brugada et al., 2012). It is also supported by the pharmacology of the channel, since the channel opener Bay K 8644 can mimic the TS arrhythmia and the channel blocker verapamil can be used to treat TS (Jacobs et al., 2006; Sicouri et al., 2007).

Kinetic analysis of *in vitro-*expressed mutant and WT versions of the TS channel reveal that the major effect of the TS mutation is to alter the speed with which the opened conducting channel returns to a non-conducting conformation through a process called channel inactivation (Antzelevitch et al., 2007; Barrett and Tsien, 2008). As would be predicted from the cardiac findings in this disorder, channel inactivation caused by changes in the membrane potential (referred to as voltage-dependent inactivation, VDI) are slowed, but there is additionally a different mechanism of inactivation regulated directly by calcium itself, and this process is greatly accelerated by the mutation. The net result of the mutant is a very rapid inactivation of half the current, and then a very slow inactivation of the remainder (Barrett and Tsien, 2008).

Induced pluripotent stem cells (iPSC) from TS patient fibroblasts (Yazawa et al., 2011) were produced and these reprogrammed cells were differentiated first into cardiomyocytes. These cells recapitulated *in vitro* the prolonged APs, irregular electrical activity and abnormal calcium signals of LQT, and roscovitine, a compound that accelerated VDI restored calcium and electrical signaling toward control. These iPSC were also differentiated into cortical neurons (Paşca et al., 2011) and they showed wide APs and increased calcium signals, similar to the cardiomyocytes. In the neurons altered patterns of calcium-dependent gene expression were observed, and some of these loci had previously been implicated in ASD. In addition, abnormal levels of the neurotransmitters norepinephrine and dopamine were observed. Like the cardiomyocytes, all of these phenotypes were reversibly corrected with roscovitine (Paşca et al., 2011). However, a different neuronal phenotype, dendrite retraction, also altered in the TS cells, was not associated with calcium permeation, but instead with ectopic activation of RhoA (Krey et al., 2013). Together these iPSC results suggest that there may be cellular phenotypes of TS of potential use in high-throughput screens for novel molecules to treat the clinical syndrome.

Whole animal phenotypes of ASD were also recapitulated with TS alleles. Heterozygous TS transgenic mice carrying a poorly expressed construct with the exon 8 G406R mutation showed abnormal behavioral phenotypes thought to model ASD. They showed altered ultrasonic vocalizations and social behavior, restricted, repetitive and perseverative behaviors, and altered responses to fear conditioning (Bader et al., 2011).

Since LQT is a hyper-excitability syndrome, it suggests that neuronal hyper-excitability is a route to ASD much as it is for seizures and epilepsy, a condition long-recognized highly co-morbid with ASD. As is discussed later, co-morbidity can most simply be seen to arise from a shared genetic architecture of these diseases, with a set of alleles and loci contributing increased susceptibility to both disorders. Since hyper-excitability is such a multifaceted perturbation of fundamental signaling mechanisms, it holds the potential of representing a core deficit in ASD, rendering it a neurobiological, rather than strictly behavioral, disorder. The recognition of such a core deficits brightens the prospect that new molecular targets can be discovered in ASD against which new generations of drugs can be developed for this disease.

## THE IMPACT OF MUTATIONS IN OTHER CALCIUM CHANNEL SUBUNITS IN ASD

While it is most straightforward to see the importance of calcium channel signaling abnormalities in autism through the lens of TS, the TS mutations clearly do not account for even a tiny fraction of cases with typical ASD. The key difference is that TS is a highly penetrant simple dominant disease with known causative single gene mutation, whereas most ASD behave as a complex multigenic disorder (Persico and Napolioni, 2013). This means that mutations contributing to typical ASD cannot be said to “cause ASD” but only to incrementally enhance susceptibility to the disease – the phenotype only being observed if a sufficient number of such contributing alleles are co-inherited, likely together with exposure to additional environmental stressors of the system. This leads to the typical inheritance pattern of a complex multigenic disorder that shows only a clustering of ASD in families, no simple segregation of the trait (Splawski et al., 2006). Another signature that shows the interaction of multiple risk alleles in developing the phenotype of ASD is that while identical (monozygotic) twins, who share 100% of their alleles, are reported up to 80–90% concordant for the disorder, dizygotic fraternal twins – genetically similar to sibs in sharing 50% of their alleles – are only about 30% concordant, but are still affected at about 20 times the general population risk (Ronald and Hoekstra, 2011). That there even is such a thing as an “environmental stressor” in the pathophysiology of ASD is demonstrated by the existence of discordant monozygotic twins who arose from the same fertilized egg and who share their entire genome in common yet have vastly different phenotypes. This discordance is a hopeful sign that therapeutics could be found to mitigate dysfunction in a susceptible genetic background. However, it will likely require a detailed understanding of the genetic architecture of this complex disease before these non-genetic stressors can be identified.

The calcium channel family is well-recognized to cause channelopathy diseases. The close paralogs of the TS locus, *CACNA1S* and *CACNA1A *have long been recognized to have highly penetrant, simple dominant mutant alleles that cause, respectively, the skeletal muscle diseases hypokalemic periodic paralysis and malignant hyperthermia (Maclennan and Zvaritch, 2011), and the neurological diseases hemiplegic migraine, episodic ataxia and spinocerebellar ataxia (Gargus, 2009; Pietrobon, 2010). Furthermore, simple dominant pathogenic mutations have also been identified in many of the accessory subunits of these channels as well.

There are, in addition, further diverse suggestions that “weak,” poorly penetrant ASD-susceptibility alleles at other calcium channel loci are germane to typical ASD (**Table 1**). First, mutations in several other calcium channel alpha subunit neuronal paralogs of the TS/LQT8 channel have been found in subjects with ASD, where they behave more like those mutations contributing to a multigenic disease described above. They do not neatly segregate with ASD in a family, but instead appear to contribute susceptibility to autism pathogenesis (Splawski et al., 2006).

**Table 1:** Calcium channels and calcium channel subunits implicated in ASD.

Protein	Description	Normal function	Disease association
CACNA1C	Voltage-regulated L-type calcium channel, alpha 1C subunit	Regulates entry of Ca^2+^ into excitable cells: muscle contraction, hormone/neurotransmitter release, gene expression, cell cycle	Timothy syndrome, ASD, psychiatric diseases
CACNA1D	Voltage-regulated calcium channel, alpha 1D subunit	High-voltage activated, long-lasting calcium activity	Sinoatrial node dysfunction and deafness, ASD, psychiatric diseases
CACNA1E	Voltage-regulated R-type calcium channel, alpha 1E subunit	High-voltage activated, rapidly inactivating	ASD, psychiatric diseases
CACNA1F	Voltage-regulated L-type calcium channel, alpha 1F subunit	Regulates entry of Ca^2+^ into excitable cells: muscle contraction, hormone/neurotransmitter release, gene expression, cell cycle	ASD and X-linked congenital stationary nightblindness
CACNA1G	Voltage-regulated T-type calcium channel, alpha 1G subunit	Regulates entry of Ca^2+^ into excitable cells: muscle contraction, hormone/neurotransmitter release, gene expression, cell cycle	ASD; intellectual disability; juvenile myoclonic epilepsy
CACNA1H	Voltage-regulated T-type calcium channel, alpha 1H subunit	Regulates neuronal and cardiac pacemaker activity	Familial autism; childhood absence epilepsy
CACNA1I	Voltage-regulated T-type calcium channel, alpha 1I subunit	Characterized by a slower activation and inactivation compared to other T-channels	Possibly implicated ASD
CACNA2D3	Voltage-regulated calcium channel, alpha 2/delta 3 subunit	Accessory calcium channel subunit; regulates entry of Ca^2+^ into excitable cells	ASD
CACNA2D4	Voltage-regulated calcium channel, alpha 2/delta 4 subunit	Accessory calcium channel subunit; regulates entry of Ca^2+^ into excitable cells	Gene deletion along with CACNA1C leads to ASD
CACNB2	Accessory calcium channel beta-2 subunit	Contributes to the function of calcium channels. Modulates voltage dependence of activation and inactivation and controls trafficking of the calcium channel family.	ASD, psychiatric diseases

The first example of such a paralog is the gene *CACNA1H. *In the families segregating mutations in this gene several cases of ASD are observed, all carrying the mutant allele, however, not all with the allele manifest diagnosable ASD. The “risk allele” simply is shown to cluster in such cases of familial autism (Splawski et al., 2006). More recently deep resequencing of functional genomic regions identified potentially causal rare variants contributing to ASD in *CACNA1F*, an X-linked gene*. *This gene was first recognized to be a locus of Stationary Night Blindness (Strom et al., 1998) but this resequencing study observed that, in addition to the eye findings, epilepsy and ASD occurred in individuals carrying gain-of-function mutations, whereas loss-of-function lesions caused only the classic Stationary Night Blindness phenotype (Myers et al., 2011). This again suggests that ASD pathogenesis arises from excess calcium signaling, but as was the case for the complex gating changes seen in TS, perhaps perturbed calcium homeostasis is more broadly responsible. *CACNA1G,* another calcium channel alpha subunit paralog, is mapped to the chromosome 17q11-q21 ASD-susceptibility region. It was found to contain single nucleotide polymorphisms (SNPs) associated with ASD in male multiplex families in an AGRE cohort (Strom et al., 2010). The same locus was again found to contain two ASD-associated SNPs in a subsequent larger study that also identified ASD-associated SNPs in *CACNA1I *and the TS locus* CACNA1C* (Lu et al., 2012). Indeed, there is even growing evidence that *CACNA1C* itself also contains other weak alleles that contribute broadly to cortical dysfunction, such as in schizophrenia, bipolar disease (BPD) and major depression (Sklar et al., 2008; Green et al., 2010; Thimm et al., 2011). In some cases mutations of this *CACNA1C* alpha subunit also alter an adjacent calcium channel accessory subunit, as in the case of an interstitial deletion at chromosome 12p13.33 that deleted both the *CACNA1C* major subunit and the *CACNA2D4* accessory calcium channel subunit genes, causing ASD-like developmental delays in two sibs and their father (Abdelmoity et al., 2011). In a subsequent study of copy number variants (CNVs) in ASD, two affected sibs were found to have a 2p:12p translocation that, again, resulted in the deletion of both genes as well (Smith et al., 2012). Eichler and Meier (2008) also identified exon-disrupting CNV deletions not found in healthy controls in the paralog calcium channel accessory subunit *CACNA2D3 *in their analysis of recurrent CNV hotspots in ASD (Girirajan et al., 2013). Eichler’s group further identified *de novo* rare alleles in alpha subunit loci *CACNA1D* and *CACNA1E *as “top *de novo* risk mutations” for autism in a whole exome resequencing study (O’Roak et al., 2012b), and, using a high density custom microarray in a different cohort of ASD, implicated a completely different type of calcium channel, a calcium-permeable cation channel called a transient receptor potential (TRP) channel, encoded by *TRPM1. *This gene had previously been shown to carry pathogenic alleles for Complete Congenital Stationary Night Blindness (Audo et al., 2009) and subsequently to participate in the metabotropic glutamate receptor signaling cascade (Devi et al., 2013). They observed that the CNV deletion at the 15q13.3 locus that is enriched in ASD, was found in 5 out of 2,588 cases, and that although *CHRNA7* had been previously implicated as contributing to neurological defects in this lesion (see below), in their cohort they found no deletions of this gene, but instead found that all five cases had deletions of *TRPM1* in this locus, including one homozygous deletion never observed in a control.

In a very powerful genome-wide association study (GWAS) of SNPs in a huge European cohort of over 30,000 cases and a similar number of matched controls, specific variants underlying genetic effects shared between the five disorders: ASD, attention deficit-hyperactivity disorder, bipolar disorder, major depressive disorder, and schizophrenia revealed that only 4 of the ~25,000 human loci were associated with neuropsychiatric disease at “genome-wide significance” – the probability of chance false positive association being less than 5 in 100 million (*p* < 5 × 10^-^^8^). Two of these associated genes encoded calcium channel subunits, *CACNA1C*, the TS locus, and the accessory calcium channel subunit *CACNB2* (Psychiatric Genomics Consortium et al., 2013). Interestingly, although no strong allele of *CACNB2 *has yet been found to cause a TS-like syndrome, dominant pathogenic loss-of-function missense alleles of the locus cause the short QT arrhythmia Brugada syndrome (Antzelevitch et al., 2007). This is the same syndrome caused by dominant loss-of-function alleles at *CACNA1C, *where a gain-of-function allele causes TS. This finding powerfully implicates the function of this multimeric calcium channel and TS-like pathophysiology in this wide spectrum of neuropsychopathology. It is also consistent with the observation that genes encoding plasma membrane calcium pumps, responsible for creating the calcium gradients dissipated by the channels, have been repeatedly associated with ASD. The calcium ATPase gene *ATP2B2* encodes the plasma membrane calcium-transporting pump which extrudes Ca^2^^+^ from cytosol into extracellular space, and three studies from different populations have reported association between this locus and ASD (Carayol et al., 2011; Prandini et al., 2012; Yang et al., 2013). It is further intriguing to note that paralogs of the other Brugada syndrome loci encoding sodium and potassium channels, also feature prominently in the architecture of ASD, as discussed below. The typical channelopathy lesions in well-understood monogenic diseases of heart, muscle and nerve cause membrane *hyper-excitability*. Hence, mutations in Ca^2^^+^ and Na^+^ channels, which physiologically excite a tissue, typically have gain-of-function lesions, while mutations in K^+^ and Cl^-^ channels, which physiologically stabilize excitable tissue, typically have pathological lesions that diminish their current (Gargus, 2008).

## SODIUM CHANNEL DEFECTS IN ASD

As mentioned above, mutations in three voltage-gated sodium channel subunits cause Brugada syndrome, the cardiac alpha subunit *SCN5A*, and accessory subunits *SCN1B *and *SCN3B. *Mutations in neuronal paralogs of the alpha subunit, *SCN1A *and* SCN2A,* had long ago been observed in rare cases of familial autism (Weiss et al., 2003) and prior to that had been shown to contribute dominant pathogenic alleles to the seizure syndrome GEFS+. *SCN1A *had also been shown to carry haploinsufficient dominant null alleles in the severe seizure syndrome SMEI/Dravet syndrome (reviewed in Ma and Gargus, 2007), as well as missense alleles in the migraine syndrome familial hemiplegic migraine (FHM3; Dichgans et al., 2005; Gargus and Tournay, 2007). Furthermore, *SCN1A* alleles have also been recognized to cause autism and epilepsy phenotypes together with biopsy-proven mitochondrial disease (Craig et al., 2012). It is particularly intriguing that the autism-associated *SCN1A* alleles are quite different from the seizure alleles, which produce a more severe lesion in the channel protein, but that they are very similar to the mutations found in the FHM3 families (Gargus and Tournay, 2007). These alleles are found to disrupt cytosolic loop domains at the C-terminus of the protein, a region originally identified in the Brugada SCN5A channel as an EF-hand-containing domain key to channel inactivation (Glaaser et al., 2006) and a site of regulatory calmodulin binding (Kim et al., 2004). Both *FHM3* and autism alleles of *SCN1A* perturb calmodulin-interacting intracellular regions of the channel protein. These regions connect this sodium channel into the calcium signaling pathways of the neuron since they interact with calmodulin that serves as an actual bound protein subunit of the channel (Gargus, 2009).

More recently strong evidence has been building for a role of lesions in neuronal voltage-activated sodium channel alpha subunits in typical polygenic ASD (**Table 2**). Whole-exome resequencing of nearly 1000 individuals uniquely identified *SCN1A* as the sole gene in which two independent probands had non-sense variants that disrupted the same gene, a highly significant result (Sanders et al., 2012) and this finding was again confirmed in a separate large resequencing study that found *de novo* protein altering mutations in the gene in probands with ASD (O’Roak et al., 2012a). Loss of function lesions in this region had previously been recognized by array-comparative genome hybridization (CGH) detection in a child with autistic features carrying a *de novo* deletion of chromosome 2q24.2-q24.3, the region containing *SCN2A *and *SCN3A* (Chen et al., 2010).

**Table 2:** Sodium channels implicated in ASD.

Protein	Description	Normal function	Disease association
SCN1A	Voltage-regulated sodium channel, type 1	Expressed in brain and muscles; involved in generation/propagation of action potentials	Familial hemiplegic migraine type 3, GEFS^+^, SMEI/Dravet syndrome, familial autism
SCN2A	Voltage-regulated sodium channel, type 2	Action potential initiation and propagation in excitable cells	Epilepsy, ASD
SCN3A	Voltage-regulated sodium channel, type 3	Action potential initiation and propagation in excitable cells	Epilepsy, ASD
SCN7A	Voltage-regulated sodium channel, type 7	Na^+^-specific channel, allowing passive flow of ions down their electrochemical gradient	Homozygous deletion in autism
SCN8A	Voltage-regulated sodium channel, type 8	Essential for the rapid membrane depolarization that occurs during the formation of the action potential in excitable neurons	Heterozygous missense mutation was linked to epilepsy and autism

Because of the fact that mutations in *SCN1A* are capable of causing seizure syndromes and seizures are frequently comorbid with ASD, the question arises whether seizure activity causes the impairments observed in ASD (Catarino et al., 2011), or if the ion channel dysfunction itself, independent of seizures, contributes to the ASD pathophysiology. This question has been elegantly approached in heterozygous KO mouse models of SMEI/Dravet syndrome. *Scn1a+/-* heterozygous KO mice develop multiple behavioral phenotypes, including increased anxiety, hyperactivity and stereotyped behaviors, in addition to seizures and ataxia (Han et al., 2012). However, using inhibitory RNA (RNAi) expression of SCN1A could be reduced in *focal* regions of the brain without producing clinical or EEG-detectable seizures (Bender et al., 2013). In this case focal loss of expression of this channel was found to cause spatial memory impairment, without an effect on response to a novel object or in more general measures of exploration. This spatial performance was significantly related to hippocampal theta frequency (an inducer of LTP, see below) in the control group, but this relationship was abolished after RNAi knockdown, consistent with a role of this channel in learning paradigms dependent upon hippocampal theta oscillations and independent of a role in seizures. This is consistent with a critical role for SCN1A in the network oscillations contributing to cognitive function (Bender et al., 2013).

Another sodium channel alpha subunit gene came to be implicated in ASD by whole-genome resequencing in a small family quartet having just one affected proband, an unaffected sib and two unaffected parents. The study uncovered yet another neuronal sodium channel paralog underlying the phenotype of autism with epilepsy. They discovered a *de novo *heterozygous missense mutation in *SCN8A* in the proband that alters an evolutionarily conserved residue in one of the most abundant sodium channels in the brain. Further, they carried out biophysical measurements of the properties of the mutant channel and demonstrated a dramatic increase in persistent sodium current and incomplete channel inactivation (Veeramah et al., 2012), demonstrating a gain-of-function lesion similar to that seen in the pathogenic cardiac and muscle sodium channel paralogs.

A different set of mechanistically unbiased approaches to ASD allele discovery have also pointed to neuronal sodium channel paralogs. The first study involved a large survey of consanguineous Middle Eastern families with autism and the technique of microarray homozygosity mapping. This technique will detect rare variants that, through common decent from a shared parental ancestor, cause recessive disease. The study identified one family that segregated a homozygous deletion of *SCN7A *(Morrow et al., 2008). This gene lies adjacent to *SCN1A* within the sodium channel gene cluster at the autism-5 locus (*AUT5*) on chromosome 2. While its mRNA is neuronally expressed, no function has yet been observed for the putative ion channel it encodes (Saleh et al., 2005). It is rapidly evolving, having arisen from *SCN1A* by endoduplication (Plummer and Meisler, 1999), and such rapidly evolving genes are a signature of genes potentially playing human-specific roles, intriguing candidates in neuropsychiatric diseases (Berglund et al., 2009).

While the Brugada syndrome associated sodium channel beta subunit locus *SCN1B* is well recognized to carry alleles that cause the GEFS+ seizure phenotype (Wallace et al., 1998) and can cause ASD together with this syndrome (Dixon-Salazar et al., 2004), no mutations in these accessory sodium channel subunits have yet been clearly associated with the ASD phenotype independent of seizures.

The sum of the evidence on neuronal sodium channels suggests that hyper-excitability-causing gain-of-function lesions that delay inactivation of the channel predominate, much as is seen in LQT and MHS. However, there are also clear cases of deletions causing haploinsufficiency, as in SMEI, and even absence of the channels, as in SCN7A. These can only be interpreted as loss-of-function lesions that, most simply, reduce membrane excitability. Again, the fact that calmodulin acts as a modulatory subunit for the alpha subunits in this family of channels, that the alpha loci carry the preponderance of the evidence for association with ASD and that the calciumopathy disease mechanism is caused by pathogenic muscle and cardiac sodium channel paralogs also serves to implicate abnormal calcium homeostasis in these neuronal lesions.

## POTASSIUM CHANNEL DEFECTS IN ASD

Calcium-activated potassium channels are central components of neuronal calcium signaling and neurosecretory pathways and critical regulators of pacemaker-like rhythmic, bursting synaptic activity, particularly involving aminergic transmitters such as dopamine. As such they were early identified candidate genes associated with neuropsychiatric phenotypes (Chandy et al., 1998; Grube et al., 2011). The large conductance (BK) family member *KCNMA1 *has more recently been implicated in ASD. Strong pathogenic alleles at this locus cause a distinctive epilepsy syndrome, “Generalized epilepsy and paroxysmal dyskinesia” (Du et al., 2005), and the locus was shown to be physically disrupted on chromosome 10 by a balanced reciprocal translocation in a patient with ASD. Following functional assessment via electrophysiology demonstrated reduced activity of the channel and confirmed its functional haploinsufficiency. Furthermore, the case-control component of the study revealed a missense allele that altered a conserved domain of the channel in another subject with ASD, with no variants detected in the control population (Laumonnier et al., 2006). More recently this BK channel was implicated in ASD because of its regulation by the fragile X mental retardation 1 protein (FMRP), the loss of which produces the monogenic ASD syndrome Fragile X (FXS), discussed further below. In presynaptic membranes FMRP was shown to physically interact with the BK channel through its regulatory KCNMB4 subunit. The loss of FMRP, via a murine *fmr1* KO lesion that models FXS, reduces channel activity, slowing membrane repolarization and broadening the AP, increasing the presynaptic calcium influx and neurotransmitter release, dysregulating synaptic transmission. This difference observed between the control and the KO animals was abolished by the intracellular calcium chelator BAPTA, suggesting that FMRP alters the calcium sensitivity of the BK channel by its effects on the channel’s regulatory subunit (Deng et al., 2013). This channel subunit was further implicated in ASD by the fact that SNPs in *KCNMB4* were strongly associated with ASD and included as one of three genes in a panel “predictive” of ASD (Skafidas et al., 2012). Another potassium channel, the A-type potassium channel encoded by *KCND2* (Gross et al., 2011) was also shown to be altered by FMRP. In this case it followed the conventional mechanism invoked for FXS pathogenesis, FMRP-sensitive synaptic protein translation downstream of the metabotropic mGluR5 glutamate receptors in the postsynaptic membrane, discussed further below. The hippocampi of *fmr1* KO animals were observed to have hyper-excitability because of reduced levels of this important repolarizing potassium channel (Gross et al., 2011). Additionally, while much attention in FXS has focused upon mGluR5 receptors and enhanced mGluR-dependent long term depression (LTD) seen in *fmr1* KO mice, FMRP binds over 400 putative mRNAs (Ashley et al., 1993) and various approaches similarly identify several other potassium channel mRNAs as FMRP targets (Lee and Jan, 2012).

Other classes of potassium channels have been implicated in ASD by rare alleles and functional studies of heterologously expressed pathogenic proteins. For example, the ATP-dependent inward rectifier potassium channel gene *KCNJ10 *(originally identified to carry pathogenic epilepsy alleles causing SESAME syndrome with ataxia, sensorineural hearing loss and tubulopathy; Bockenhauer et al., 2009), was identified to carry missense mutations that altered highly conserved residues in two unrelated families with seizures and ASD (Sicca et al., 2011). The effects of mutations in a heterologous expression system revealed an increase in channel current suggesting a gain-of-function defect (Sicca et al., 2011). The M-current potassium channel subunit gene *KCNQ3* (paralog of the first LQT gene and long-proven causal of Benign Familial Infantile Seizures; Wang et al., 1998) and its paralog encoding one of its heteromultimeric partners, *KCNQ5*, have also been recently implicated in ASD. A *de novo* translocation truncating *KCNQ3 *was identified in a boy with autism, and three patients with ASD were recognized to share the same rare variant of *KCNQ5 *caused by a SNP that creates a missense mutation that could be proven to be loss-of-function by patch clamp recording of oocytes co-expressing both subunits (Gilling et al., 2013). Potassium channels implicated in ASD are summarized in **Table 3**.

**Table 3:** Potassium channels and potassium channel subunits implicated in ASD.

Protein	Description	Normal function	Disease association
KCNMA1	Calcium-activated large conductance potassium channel, subfamily A	Both voltage-and calcium-sensing channel, controls smooth muscle tone and neuronal excitability	Epilepsy (GEPD); implicated in ASD
KCNMB4	BK-channel beta subunit	Predominantly expressed in neuronal tissue. Changes voltage dependence and activation kinetics of KCNMA1	ASD
KCNJ10	ATP-sensitive inward rectifier potassium channel 10	Have a greater tendency to allow potassium to flow into, not outside of the cell	Seizures, ataxia, and ASD
KCNQ3	Potassium voltage-gated channel (M-channel)	Slowly activating and deactivating channel, plays a role in the regulation of neuronal excitability	Seizures and ASD
KCNQ5	Potassium voltage-gated channel (M-channel)	Activates slowly with depolarization and expressed in subregions of the brain and skeletal muscle. Can multimerize with KCNQ3	Implicated in ASD
KCND2	Potassium voltage-gated channel (A-channel)	Mediates a rapidly inactivating outward K^+^ current in neurons and the heart	Implicated in ASD

## CHLORIDE CHANNEL DEFECTS IN ASD

The balance between excitatory and inhibitory neurotransmission (E/I balance) has long been recognized to play a critical role in the development of neuronal circuits, the maturation of the brain and to be impaired in several genetic and teratological models of ASD (Eichler and Meier, 2008; Gibson et al., 2009; Gogolla et al., 2009; Pizzarelli and Cherubini, 2011,^2013^; Bateup et al., 2013; Lin et al., 2013). Just as glutamate is the major excitatory neurotransmitter in the CNS, gamma aminiobutyric acid (GABA), produced from glutamate, is its major inhibitory neurotransmitter, and an imbalance in the signaling of these two systems is implicated in ASD (Gogolla et al., 2009). GABA’s actions are mediated through the GABA A- and B- type receptors. The metabotropic GABA-B receptors are GPCRs that function by increasing the potassium conductance responsible for long-lasting inhibitory postsynaptic potentials (Kuriyama et al., 2000). GABA-A receptors, on the other hand, are heteropentamer channels comprised of two α-, two β- and one γ- or δ-subunit (Wafford, 2005; Goetz et al., 2007). The GABA-A receptors consist of at least 15 different receptor subunits, and mutations in GABA-A receptor subunits have been frequently associated with epilepsy and other neuropsychiatric disorders (Kang and Barnes, 2013). They form a ligand-gated chloride channel that plays a crucial role in inhibitory synaptic transmission. It is able to play this role since the chloride Nernst potential is near the resting membrane potential of most cells, including neurons, and opening this channel serves to anchor the potential, preventing a depolarizing AP. It has more recently, however, been observed that GABA can actually play the role of an *excitatory* *depolarizing* neurotransmitter early in development and it does so because the intracellular chloride ion concentration in the target neuron is elevated, rendering its Nernst potential a depolarizing force, similar to the activation of a sodium channel. This elevated intracellular chloride arises because of high activity of the loop diuretic-sensitive Na-K-Cl-Cl electroneutral cotransporter found ubiquitously expressed in plasma membranes, encoded by *SLC12A2*. It drives chloride into the cell making use of energy provided by the inward sodium gradient. This cotransporter is the paralog of the kidney-specific apical membrane cotransporter mutated in Bartter syndrome, encoded by *SLC12A1, *through which bumetanide and furosemide achieve their diuretic effect. Concomitantly through development, activity of the chloride exporting K-Cl electroneutral cotransporter, encoded by *SLC12A5*, and powered by the outward potassium gradient, is decreased. This coordinate regulation of the two cotransporters is mediated by opposite-acting protein phosphorylation events on each cotransporter via activation of WNK serine-threonine protein kinase (Ben-Ari et al., 2012). In KO mice lacking SLC12A5 protein, unlike WT, patch-clamp measurements demonstrated an excitatory action of GABA (as well as another neurotransmitter that activates chloride channels, glycine), implicating the altered chloride gradient in this shift (Hübner et al., 2001). This has led to the suggestion that perhaps the E/I balance problem in ASD arises from GABA continuing to have an “immature” activating effect at its GABA-A receptors because of an abnormal chloride gradient. The conclusion might then be that bumetanide could remedy that situation, restoring the gradient and GABA inhibition and thereby prove therapeutic in ASD. A small double-blinded clinical trial of 60 children showed some efficacy on a number of ASD assessment scales during a 3-month trial (Lemonnier et al., 2012).

## OTHER SODIUM-COTRANSPORTERS INVOLVED IN CALCIUM SIGNALING IN ASD

The sodium-dependent solute transporters, introduced above, are a large family of transmembrane carrier proteins. They are able to function as secondary active transporters, moving solutes against their electrochemical gradient into or out of cells, by tapping the energy stored in the transmembrane sodium gradient. The aminergic synapses rely upon members of this family to regulate synaptic transmission by clearing neurotransmitters that were released into the cleft during synaptic transmission, and mutations in these important proteins have been observed in ASD. Two ASD subjects with CNV duplications of *SLC1A1*, encoding the sodium-potassium dependent EAAT glutamate transporter found widely distributed in the brain, were observed in a large survey of CNV hotspots in ASD (Girirajan et al., 2013). A common polymorphism was also observed in *SLC6A4 *to be associated with ASD (**Table 4**). It encodes the high-affinity sodium-chloride dependent serotonin transporter (5-HTT) localized in brain presynaptic neuronal membranes (Li et al., 2012). This transporter is a principal site of action of tricyclic antidepressants and serotonin re-uptake inhibitors. A polymorphism in the promoter region located 1 kb upstream of the transcription initiation site is composed of repeat elements and the polymorphism is a 44-bp indel with the short allele associated with lower transcriptional efficiency. Association studies of autism with this polymorphism have yielded conflicting results, but a recent large meta-analysis of all studies failed to find a significant overall association between either polymorphism and autism (Huang and Santangelo, 2008).

**Table 4:** Transmembrane receptor genes implicated in ASD.

Protein	Description	Normal function	Disease association
*ATP2B2*	Ca-transporting plasma membrane ATPase	pumps Ca out of the cell into extracellular space	Hearing loss, ASD
*CADPS2*	Calcium-dependent activator of secretion	Calcium-binding protein, regulates exocytosis of synaptic vesicles	ASD
CHRNA7	Ligand-gated cation channel	Regulates glutamate release presynaptically and stimulates inhibitory GABAergic interneuron activity postsynaptically	Epilepsy, schizophrenia, speech and learning problems
GABRG3	GABA-A gamma subunit of GABA-receptor family	Conducts chloride ions upon activation, leading to hyperpolarization. Causes inhibitory effect on neurotransmission	SNPs associated with ASD
SLC1A1	Solute carrier, glutamate transporter	Postsynaptic protein, help terminating action of the neurotransmitter glutamate. Also transports aspartate	Schizophrenia, ASD
SLC6A4	Solute carrier, serotonin transporter	Clears neurotransmitter serotonin from synaptic space, carrying it into presynaptic neurons.	Bipolar disorder, depression, obsessive-compulsive disorder, ASD
SLC12A2	Solute carrier, Na-K-Cl-Cl electroneutral cotransporter	Drives chloride into the cell. Ubiquitously expressed in many cell types, including cortical neurons	ASD
SLC25A12	Solute carrier, mitochondrial aspartate-glutamate carrier	Transports aspartate from mitochondria to cytosol in exchange for glutamate	ASD
TRPM1	Transient receptor potential channel	Ca2^+^ - permeable cation channel	Congenital stationary night blindness, ASD

It is intriguing to note that another ASD-associated CNV duplication was found in a membrane protein participating at the opposite end of the neurosecretion signaling pathway – the exocytosis of vesicles filled with neurotransmitters. Duplications of *CADPS2*, a gene encoding the Ca^2^^+^-dependent activator protein for secretion 2, a calcium-binding protein found in neurons involved in the exocytosis of dense-core neurotransmitter vesicles, was found in subjects with ASD (Girirajan et al., 2013). This gene is located on chromosome 7q31.32 within the *AUTS1* locus that has been previously implicated in autism susceptibility. Alternative splicing with exon 3 skipping was observed in subjects with autism with no splice variants found in healthy controls (Sadakata et al., 2007). A mouse model with exon 3 skipping demonstrated decreased reciprocal interactions, increased anxiety and decreased exploratory behavior in open-field tests, as well as maternal neglect of newborns and altered ultrasound vocalization (Sadakata et al., 2012).

Members of the solute carrier superfamily also mediate transport across the mitochondrial inner membrane. Several studies in ASD have focused attention on mitochondrial dysfunction (Gargus and Imtiaz, 2008) and linkage and association studies have further focused upon variation in *SLC25A12 *(Turunen et al., 2008; Kim et al., 2011; Napolioni et al., 2011). This gene on 2q24 encodes the brain-specific isoform of the mitochondrial calcium-regulated aspartate/glutamate carrier. Candidate genes in this region were scanned for autism-associated SNPs and two, located in introns of *SLC25A12*, were found associated with the disease (Ramoz et al., 2004). The same risk haplotype at these two SNPs was then confirmed to be linked and associated with ASD in a second cohort (Ramoz et al., 2004). Subsequent studies confirmed that autism was associated with other SNPs within the locus, although none appeared to alter function (Segurado et al., 2005). Subsequently a study of post-mortem brain tissue showed significantly increased transport activity by the SLC25A12 transporter in subjects with ASD. However, no mutations or polymorphisms were found associated with the disease (Palmieri et al., 2010). Furthermore, all of the excess enzyme activity found in brain samples from patients with ASD was calcium-dependent and was found to be associated with elevated cytosolic calcium levels in these subjects (Palmieri et al., 2010). They found that controlling for the calcium levels, transport activity was identical in isolated mitochondria from patients and controls. They therefore concluded that the critical link to this altered mitochondrial metabolism observed in the brains of patients with autism was in fact caused by altered calcium homeostasis, although it was never directly studied. Subsequent studies have found overexpression of *SLC25A12* in post-mortem brain from subjects with ASD (Lepagnol-Bestel et al., 2008). While the fundamental abnormality of this transporter in ASD remains to be unambiguously defined, it is a tantalizing link between cytosolic calcium homeostasis and mitochondrial energetics, an often independent important mechanistic theme in ASD etiology (Gargus, 2010).

## SYNAPSES AND DOWNSTREAM CALCIUM SIGNALING DEFECTS IN ASD

The axonal AP-induced calcium signal culminates by initiating the fusion of synaptic vesicles into the pre-synaptic membrane, and in this fashion participates in diverse mechanisms of synaptic modulation that produce synaptic plasticity and learning (Neher and Sakaba, 2008). Crossing the synaptic cleft, the neurotransmitter binds and activates its receptor, altering excitability of the post-synaptic cell. It also often feeds back through pre-synaptic receptors to modulate pre-synaptic vesicle fusion. Ultimately the signal is terminated by re-uptake of the neurotransmitter from the synaptic cleft. Calcium not only plays a role in neuronal plasticity in modulating neurosecretion in neuronal networks, but it additionally shapes the composition of the synaptic membranes themselves through the role it plays in the calcium-sensitive mammalian target of rapamycin (mTOR) signaling pathway of upstream regulators and downstream effectors, many directly implicated in model monogenic ASD syndromes.

## GLUTAMATE RECEPTORS

Glutamate, the major excitatory neurotransmitter in the central nervous system, activates two major classes of synaptic receptors: ionotropic receptors, which are themselves ligand-gated cation channels, and metabotropic receptors, that are heptahelical GPCR coupled to a variety of signaling pathways through trimeric G proteins. mGluR5, *GRM5*-encoded receptors, are coupled via Gq to activate phospholipase C and in turn an IP3 and the calcium signaling second messenger system. Phosphorylation and dephosphorylation of a key residue within the C-terminal domain of the activated receptor cause synchronous, oscillatory changes in IP3 and Ca^2+^ levels (Bradley and Challiss, 2011).

The metabotropic glutamate receptor mGluR5 is one of the many synaptic proteins altered in *Fmr1* KO mice. FXS is an important monogenic model of ASD (Iossifov et al., 2012), and mouse models of FXS are based upon such knock-out (KO) mice. In the absence of FMRP, it is widely recognized that performance in learning paradigms is impaired in these mice and that this recapitulates the phenotypes observed in FXS patients where defects in learning are directly observed (De Rubeis et al., 2012). The absence of FMRP in *Fmr1* KO mice produces an up-regulation of mGluR5 and an enhancement in the synaptic phenomenon of LTD that relies upon enhanced mTOR-mediated rapamycin-sensitive protein synthesis triggered by this receptor. This culminates in reduction of synaptic AMPA glutamate receptors (see below) and chronically decreased synaptic efficiency (Sharma et al., 2010).

Fragile X syndrome is a loss-of-function syndrome, overwhelmingly caused by an extremely large expansion of the trinucleotide CGG repeats (>200) in the 5′ non-coding portion of the human locus, but is also caused by deletion of the locus or rare missense mutations (Collins et al., 2010). Males are predominantly affected, and this lesion typically results in the absence of FMRP, a multi-functional mRNA binding protein (Ashley et al., 1993) and ion channel regulatory subunit (Deng et al., 2013).

Carriers of one *FMR1 *allele with a “premutation,” a modestly expanded trinucleotide repeat (55–200 repeats) at the *FMR1 *locus, are at risk for expression of “Fragile X-associated tremor/ataxia syndrome” (FXTAS), an aged-onset monogenic neurodegenerative disorder associated with decreased FMRP levels (Feng et al., 1995; Jacquemont et al., 2003; Sheridan et al., 2011; Cao et al., 2013). iPS-derrived neurons harboring a stably active, modestly expanded allele have reduced neurite length and high amplitude, high frequency functionally abnormal calcium transients compared to neurons harboring the normal active allele (Liu et al., 2012). Moreover, a sustained calcium elevation was found in the expanded-allele-expressing neurons after glutamate application (Liu et al., 2012). Subsequently astrocytes derived from heterozygous mice with the FMRP premutation also demonstrated increased Ca^2^^+^ oscillations as well as increased sensitivity to glutamate, despite having levels of mGluR5 receptors similar to control (Cao et al., 2013). Comparable studies were not reported with classical FXS alleles, however, these observations suggest a fundamental role of FMRP in synaptic calcium signaling that is sensitive to disruption by pathogenic alleles at the locus and is potentially consistent with the effects of FMRP observed on pre-synaptic BK channels discussed above (Deng et al., 2013).

The postsynaptic membrane mGluR5 receptors are bound to HOMER1 scaffold proteins, together with plasma membrane *GRM1*-encoded mGluR1 receptors, ER-associated IP3 and RyR, encoded by *ITPR1, RYR1 *and* RYR2,* and plasma membrane voltage dependent calcium channels, to form a functional signaling complex. Together with SHANK1 and SHANK3 scaffolds it plays a key role in facilitating intracellular calcium release and transmembrane calcium currents (Kato et al., 2012). Interestingly, in *Fmr1* KO mice disrupted Homer1 complex formation is observed (Ronesi et al., 2012). There are two isoforms of Homer1, long and short, that have opposite actions in signaling. The long form has a coiled-coil domain through which they multimerize to act as a scaffold. The N-terminal domain of the long form binds to the intracellular C-terminal tail of the mGluR5 receptor and has an ability to link them together with the IP3 receptors and other scaffolded signaling membrane proteins and kinases (Ronesi et al., 2012). Homer1 has several splice variants, and extracellular stimuli promote 3′-end processing of Homer1 pre-mRNA, leading to the switch of poly(A) site selection. The short form of Homer1, an immediate early gene transcript, lacks this coiled-coil domain, and thus cannot multimerize or act as a scaffold. It therefore serves to physically dissociate the receptor from the IP3 receptor and other scaffolded proteins. This renders the mGluR5 receptor constitutively active and agonist-independent, enhancing its signaling. In *Fmr1* KO mice mGluR5 is preferentially associated with the short form of Homer1. This leads to an increased signaling frequency and prolonged spontaneous persistent activity. Genetic deletion of short-form Homer1 corrects several phenotypes in *Fmr1* KO mice, while a short mimic peptide containing the proline-rich motif of mGluR5 that binds to Homer and disrupts the mGluR5-Homer scaffold, in wild type mice produces several phenotypes of *Fmr1* KO mice. Together it suggests an important role of mGluR5-long Homer signaling complexes in normal brain function and of the short form complexes in the disease pathophysiology. In fact, pharmacological inhibition of mGluR5 activity was proven beneficial in correction of autistic phenotypes in both animal models and human patients, probably because of its effect on the constitutively active mGluR5 (Michalon et al., 2012; Ronesi et al., 2012).

Calcium signaling through metabotropic mGluR5 receptors additionally plays a key role in modulating the function and subunit composition of ionotropic glutamate receptors at the synapse. *N*-methyl-D-aspartate (NMDA) inotropic glutamate receptors are a heterotetramer of subunits encoded by *GRIN1 *and* GRIN2A, B, C or D*. The switch of subunits in the NMDA receptor from GRIN2B to GRIN2A is an important example of synaptic modulation and experience-dependent regulation of receptor subunit composition *in vivo*. This long-lasting alteration of the synapse is driven acutely by activity of mGluR5 and involves its downstream phospholipase C, calcium release from IP3-dependent stores, and protein kinase C activity. In mGluR5 KO mice the developmental switch is deficient and therefore the GRIN2B to GRIN2A switch evoked *in vivo* by visual experience is absent and such learning fails (Matta et al., 2011). The subunit switch causes important changes to NMDA receptor function, altering the amount of calcium influx through the ionotropic receptor channel pore and the types of proteins interacting with the intracellular domain of the receptor. These features regulate the type of long-term synaptic plasticity (LTP or LTD, see below) that NMDA receptor activation can induce (Liu et al., 2004; Bartlett et al., 2007).

Long-term potentiation (LTP), the opposite of LTD, can be induced by rapid theta burst stimulation (TBS) in hippocampal slices and it relies upon two classes of iontropic glutamate receptors, the NMDA receptors and the alpha-amino-3-hydroxy-5-methyl-4-isoxazole propionate (AMPA) receptors, a heterotetramer with subunits encoded by *GRIA1–4* that function cooperatively as ligand-gated cation channels in the post-synaptic membrane. The AMPA subunit encoded by *GRIA2* is subject to RNA editing (CAG->CGG; changing a single amino acid within the second transmembrane domain from glutamine to arginine) which renders the channel impermeable to Ca^2^^+^, a feature essential to its function as a non-selective monovalent cation channel (Barbon and Barlati, 2011). On the other hand the NMDA receptor channel is highly calcium permeable. However, it has a complex double-gated mechanism that requires *both *binding the activating glutamate neurotransmitter and a strong membrane depolarization, usually driven by the AMPA receptors, to remove a luminal Mg^2^^+^ion that blocks the receptor’s channel (Ogden and Traynelis, 2011). Only a very large synaptic glutamate release activates sufficient AMPA channels to remove the Mg^2^^+^ ion block to provide an NMDA calcium conductance pathway and hence provide a post-synaptic calcium signal. LTP depends upon this change in postsynaptic calcium since intracellular injection of the calcium chelator EGTA blocks the development of LTP (Lynch et al., 1983). NMDA-mediated responses are required to induce LTP but it is through calcium-dependent modification of the postsynaptic neuron AMPA receptor components that LTP is manifest (Muller et al., 1988).

Loss of FMRP also leads to changes in the synaptic phenomenon of short term potentiation (STP) whereby prior activity of a synapse enhances the probability of synaptic vesicle release with a subsequent stimulation a short while later. This is largely a result of residual elevated calcium levels from the pre-synaptic priming stimulus (Fioravante and Regehr, 2011). Like LTP, STP is considered to be one of the elemental components of information processing at a synapse underlying plasticity and learning as it precedes the development of LTP. Loss of FMRP leads to enhanced responses to high-frequency stimulation and to abnormal excessive enhancement of synaptic processing of natural stimulus trains. These changes are associated with exaggerated calcium influx in presynaptic neurons during high-frequency stimulation, enhanced synaptic vesicle recycling, and enlarged readily releasable and reserved vesicle pools, all serving to increase signaling across the synapse (Deng et al., 2011).

Hence results of the loss of FMRP are changes in pre-synaptic and post-synaptic channel function, neurosecretory dysfunction and changes in the expression of a large set of post-synaptic neuronal mRNAs, many themselves changing the composition of the post-synaptic membrane. Therefore, while FXS is a simple monogenic disease it produces complex polygenic dysregulation of the synapse (De Rubeis et al., 2012). However, the sum of the evidence is that the complex pre- and post-synaptic membrane changes recognized in FXS participate in core fundamental processes of synaptic plasticity and learning, are calcium sensitive, and as such this fundamental physiological process may be a promising target in ASD amenable to pharmacological treatment.

## THE CYS-LOOP FAMILY OF nACh AND GABA RECEPTORS

The 15q11-13 inverted duplication remains the most common chromosomal lesion in ASD, and ASD-associated CNV duplications and deletions in this region are among the most common as well. This region spans the imprinted Angelman/Prader-Willi region, and both Angelman syndrome (AS) and Prader-Willi syndrome (PWS) are themselves important single locus models of ASD (Vorstman et al., 2006; Depienne et al., 2009; Dykens et al., 2011). This region contains a cluster of inhibitory GABA-A receptor subunit genes as well as the surprisingly related excitatory α7 nicotinic acetylcholine receptor (nAChR) encoded by *CHRNA7*. Both are members of the Cys-loop ligand-gated ion channel superfamily. The GABA receptors contribute to a chloride channel and the α7 nACh receptor is a homo-pentameric ligand-gated calcium-conducting cation channel. In the hippocampus, presynaptic α7 nAChRs regulate glutamate release, whereas postsynaptic α7 nAChRs stimulate inhibitory GABAergic interneuron activity (Johnstone et al., 2011) and the release of GABA (Adams et al., 2012).

Chromosomal mapping studies have revealed that several of the GABA-A receptor subunit genes are organized as clusters and one such cluster, which consists of the GABA-A receptor β3 (*GABRB3*), α5 (*GABRA5*) and γ3 (*GABRG3*) subunit genes, is located at 15q12 (Menold et al., 2001). Two SNPs located within *GABRG3* show significant evidence for linkage disequilibrium (LD) with ASD, suggesting that it or a gene nearby contributes to genetic risk in ASD (Menold et al., 2001). A small sample of subjects with ASD, studied with positron emission tomography (PET) using a benzodiazepine receptor selective PET ligand, recently provided preliminary direct evidence of changes in the abundance of the α5 GABA-A receptor in two regions of the brain, the amygdala and nucleus accumbens, limbic system structures long implicated in ASD (Mendez et al., 2013).

The molecular defects underlying AS are heterogeneous – most commonly caused by large *maternal* deletions of chromosome 15q11-q13 – but importantly, over a dozen loss-of-function mutations of *UBE3A* cause AS, implicating it as the critical 15q locus for this syndrome. In contrast to AS, PWS is caused by the absence of expression of the *paternally* active genes in this critical region of 15q11-13, and while it is uncertain which gene is critical, it is clear that its maternal allele is virtually inactive through imprinting (Chamberlain and Lalande, 2010). Importantly more than 99% of individuals with PWS have a diagnostic abnormality in the parent-specific methylation imprint within the Prader-Willi critical region (Cassidy et al., 2012) and there is clear evidence that such imprinted genes in this region play a role in ASD-like phenotypes (Chamberlain and Lalande, 2010; Relkovic et al., 2010; Ingason et al., 2011).

Interestingly deletion of just the small Prader-Willi imprinting center (PWS-IC) within 15q11-13 disrupts long-range imprinted gene expression resulting in PWS (Yasui et al., 2011), and the two PWS-IC sites flank *CHRNA7*. Recently, four probands were identified with small deletions in 15q13 that included only the *CHRNA7 *gene, and this was followed by the identification of others also with isolated heterozygous *CHRNA7* gene deletions, including the first *de novo* deletion and one patient homozygous for the deletion. These patients demonstrated the similar wide range of ASD phenotypic features associated with the larger 15q11-13 microdeletions, suggesting *CHRNA7* was the critical gene responsible for the clinical findings associated with the 15q13 microdeletion syndrome (Hoppman-Chaney et al., 2013).

A physical interaction between the DNA *cis* PWS-IC regulatory elements that flank *CHRNA7* and the protein *trans* regulatory element methyl CpG binding protein 2, encoded by *MeCP2*, is required for optimal expression of AS/PWS region genes implicated in the ASD phenotype (Yasui et al., 2011). MECP2 acts as a calcium-dependent transcriptional repressor for methylated genes, a global regulator of histone function (Cohen et al., 2011; Li et al., 2011) and plays a key role in the control of neuronal activity-dependent gene regulation. Rett syndrome is an X-linked dominant disorder caused by *MeCP2* mutations that are lethal in hemizygous males and cause an ASD-like syndrome in heterozygous females. Patients with Rett syndrome or even those with typical ASD, revealed significantly reduced *CHRNA7* expression in the frontal cortex compared with controls, suggesting that transcription of *CHRNA7* is modulated by these regulatory elements and is involved in ASD-like phenotypes (Yasui et al., 2011). In heterozygous KO mice, reduced *Chrna7* expression results in decreased hippocampal α7 receptor density, abnormal hippocampal auditory sensory processing, and increased hippocampal pyramidal neuron activity, together demonstrating that decreased receptor expression alters hippocampal inhibitory circuit function. This was associated with reduced levels of the enzyme responsible for converting glutamate to GABA, glutamic acid decarboxylase 1 (encoded by *Gad1*), and with a decrease in the GABA-A receptors, the later only observed in the males, an intriguing finding in a target for a disease as highly sexually dimorphic as ASD (Adams et al., 2012).

In *Mecp2 *KO mouse brain, the mRNA expression and protein levels of *Ube3a*, the target in AS, are decreased, and the non-imprinted adjacent GABA-A receptor *GABRB3* gene also shows reduced expression (Samaco et al., 2005). Direct study of neuronal function in the mouse *Mecp2* KO Rett model revealed that excitatory, but not inhibitory, synapses showed less spontaneous activity than control. This observation suggests a potential defect in the calcium-dependent processes of excitatory neurosecretion and synaptic vesicle trafficking (Nelson et al., 2006). Loss of MeCP2 and doubling of MeCP2 dosage in mice have opposite effects on excitatory synapse numbers in individual neurons (Chao et al., 2007), suggesting that MeCP2 may be a rate-limiting factor in regulating glutamatergic synapse formation, and this is recapitulated in neurons derived from iPSCs. Neurons derived from iPSC clones carrying 3 different MeCP2 mutations (RTT) showed a reduction in the density of glutamatergic synapse markers when compared to WT control, and neurons derived from WT iPSCs expressing an antisense knock-down construct of MeCP2 (shMeCP2) showed a similar reduction when compared to control neurons expressing a scrambled control. Finally, overexpression of MeCP2, using a lentiviral vector, increased abundance of the glutamatergic marker in WT and RTT neurons, together strongly suggesting that MeCP2 is a rate-limiting factor regulating glutamatergic synapse number in human neurons (Marchetto et al., 2010).

A disturbance in neuronal calcium homeostasis is also observed in *Mecp2 *KO mice (Mironov et al., 2009), and this phenotype too is recapitulated in iPSC-derived RTT neurons. While both WT and RTT neurons showed similar APs and sodium and potassium currents in response to depolarizations in this model (but not in other RTT models; Farra et al., 2012), demonstrating that RTT cells are not altered in maturation toward normal electrophysiological activity, spontaneous calcium transients were decreased in the RTT neurons and the frequency of calcium oscillations in RTT neurons and in WT neurons expressing shMeCP2 was abnormally decreased compared to controls (Marchetto et al., 2010). These spontaneous calcium transients could be blocked with the sodium channel blocker tetrodotoxin (TTX) or with AMPA and NMDA glutamate receptor antagonists, and were increased by GABA-A receptor antagonists, demonstrating the sensitivity of this calcium signal to synaptic activity and the presence of glutamatergic and GABAergic synapses in the system. Such neuronal activity-induced calcium influx can trigger the calcium/calmodulin-dependent protein kinase (CamK), an inducer of MeCP2 phosphorylation.

The importance of GABAergic transmission in these ASD phenotypes is revealed by the study of KO mice lacking MeCP2 selectively only from GABA-releasing neurons. They recapitulate numerous Rett syndrome and ASD phenotypic features, including repetitive behaviors. Furthermore, loss of MeCP2 from just a subset of forebrain GABAergic inhibitory neurons also recapitulates many features of Rett syndrome (Chao et al., 2010). MeCP2-deficient GABAergic neurons show reduced inhibitory quantal size, suggesting less neurotransmitter per synaptic vesicle. This finding is consistent with the observed presynaptic reduction in the GABA-synthesizing enzymes glutamic acid decarboxylase 1 (Gad1) and glutamic acid decarboxylase 2 (Gad2), and decreased GABA immunoreactivity. The pattern is similar to what is observed in the *Chrna7 *KO mouse. Together this suggests that *MeCP2* and *CHRNA7* expression is critical for normal function of GABA-releasing inhibitory neurons and that this may contribute to the neuropsychiatric phenotypes in Rett, other monogenic models of ASD, and perhaps more typical ASD (Chao et al., 2010; Yasui et al., 2011). Together these findings link gene dysregulation in the mammalian brain within the chromosome 15q11-q13 region with MeCP2 function, and therefore link Rett syndrome, AS, PWS and ASD, and suggest that ASD may be caused by the inability of neurons to generate adaptive responses via the neuroreceptors involved in generating synaptic calcium signals and calcium-regulated gene expression (Qiu and Cheng, 2010).

Finally, it is intriguing that the *combined *modulation of the two different “Cys-loop” superfamily receptors encoded in the AS/PWS region, the α7 nAChR and α5 GABA-AR, alter hippocampal function in learning paradigms. Transient application of two separate allosteric modulators, which individually inhibit either the inhibitory α5 GABA-ARs or enhance the activating α7 nAChRs, only jointly causes LTP of induced excitatory postsynaptic currents (EPSCs) in pyramidal neurons of rat hippocampal slices (Johnstone et al., 2011). Remarkably this effect is replicated by a single compound that was designed to simultaneously carry out both activities specifically on these two related receptors, suggesting the therapeutic utility of this strategy targeting the AS/PWS encoded receptors (Johnstone et al., 2011).

## mTOR SIGNALING

**FIGURE 2:**
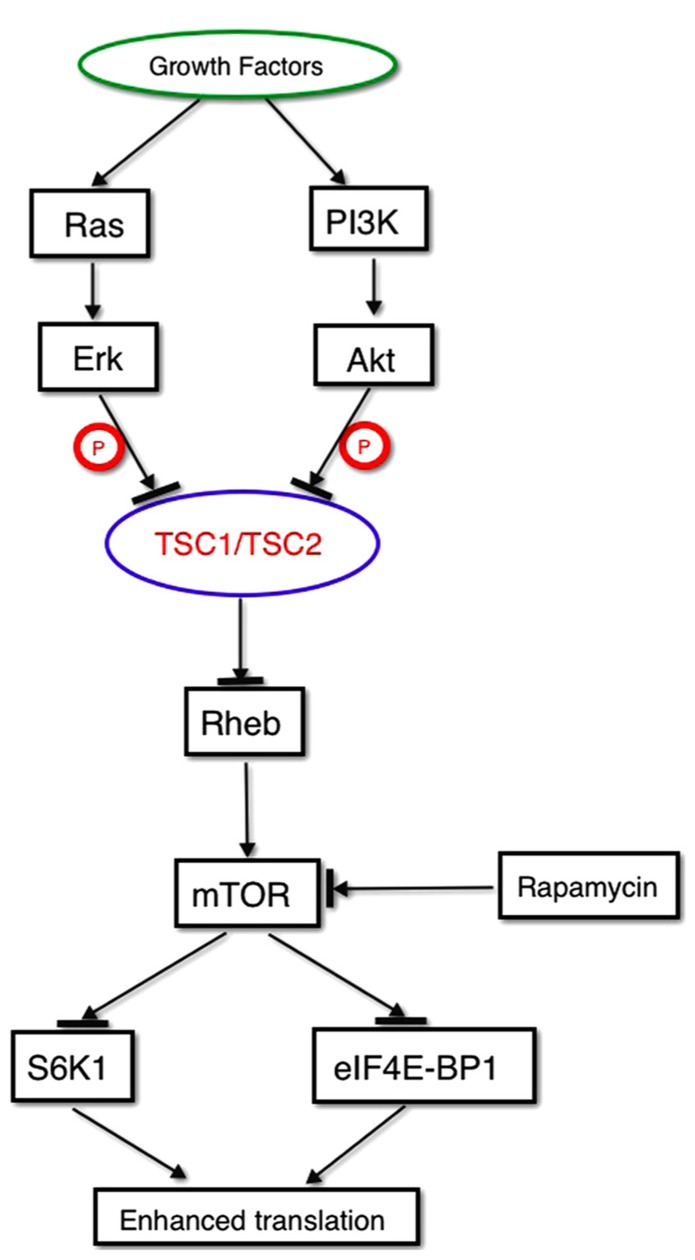
**mTOR signaling**.

Mammalian target of rapamycin is a key cytosolic integrative regulator of calcium signaling and mitochondrial function created by a large multidomain protein kinase that regulates cell growth and metabolism in response to environmental signals (Ramanathan and Schreiber, 2009) and several forms of synaptic plasticity (Hoeffer and Klann, 2010; **Figure 2**). Upstream signals originate from plasma membrane growth factor receptors that signal through phosphatidylinositol 3-kinase (PI3K) to Akt, or through Ras to ERK, to the TSC1/TSC2 heteromultimer that sits at the center of this growth factor receptor stimulated calcium signaling pathway. The genes that encode these two subunits, *TSC1* and *TSC2, *carry dominant mutations that produce tuberous sclerosis (TSC), another important syndromic form of ASD that impacts synaptic calcium signaling. The protein products of these two *TSC* genes heteromultimerize to negatively regulate downstream signaling by acting as a GTPase-activating protein (GAP) for the small GTPase RHEB, a direct activator of the protein kinase activity of mTOR. mTOR itself phosphorylates S6 kinase 1 (S6K1) and eukaryotic translation initiation factor 4E binding protein 1 (eIF4E-BP1), leading to enhanced protein translation (Ehninger and Silva, 2011).

Tuberous sclerosis is a neurocutaneous syndrome that produces ASD-like behaviors, seizures, intellectual disability, tumorous growths in the brain and characteristic skin lesions. In a TSC mouse model created by an* in vivo *postnatal *Tsc1* conditional KO in only the CA-1 hippocampal neurons, hippocampal mGluR-LTD was abolished, whereas a protein synthesis-independent form of NMDA receptor-dependent LTD (see above) was preserved (Bateup et al., 2011). Additionally, AMPA and NMDA receptor-mediated EPSCs and miniature spontaneous EPSC frequency were enhanced in *Tsc1* KO neurons. These changes in synaptic function occurred in the absence of alterations in presynaptic transmitter release probability, indicating that signaling through Tsc1/2 is required for the expression of specific forms of hippocampal synaptic plasticity and normal excitatory synaptic function (Bateup et al., 2011).

In a different mouse model of TSC, both heterozygous and homozygous loss of *Tsc1* was limited to principal cells (PC) in the cerebellum, a region of the brain only recently implicated in the ASD phenotype (Tsai et al., 2012). These KO lesions decrease PC excitability and also result in autistic-like behaviors, including abnormal social interaction, repetitive behavior and vocalizations. Importantly, treatment of these mutant mice with the mTOR inhibitor rapamycin prevented the pathological and behavioral deficits, defining a molecular basis for a cerebellar contribution to ASD (Tsai et al., 2012).

The FKBP rapamycin-binding subunit of mTOR (encoded by *Fkbp1A*;**often called *Fkbp12*) is the validated target of rapamycin and other immunosuppressant and anticancer drugs. The protein is a cis-trans prolyl isomerase that interacts with intracellular calcium release channels. Neuronal deletion of *Fkbp1A *is associated with disinhibited mTOR signaling and altered synaptic plasticity, and memory (Hoeffer et al., 2008). The KO mouse shows increased basal mTOR and S6K1 phosphorylation as well as an enhancement in hippocampal LTP. Not unexpectedly, this enhancement was resistant to rapamycin, but not to other blockers of protein translation. This conditional KO displayed enhanced contextual fear memory and autistic/obsessive-compulsive-like perseveration in several assays, together indicating that FKBP plays a critical role in the regulation of mTOR, LTP, memory, and ASD-like behaviors (Hoeffer et al., 2008). Disrupting* Fkbp1B* with interfering RNA (RNAi) knockdown also destabilizes Ca^2^^+^ homeostasis in hippocampal neurons, similarly to rapamycin, and is sufficient to induce a characteristic “aged phenotype” of Ca^2^^+^ dysregulation in even young rodents. This phenotype, seen in very old animals and Alzheimer models (Gant et al., 2011), derives from abnormal calcium signaling in hippocampal pyramidal neurons seen as increases in several Ca^2^^+^-dependent phenomena (calcium transients, slow after hyperpolarizations, voltage-gated calcium channel activity, ryanodine receptor calcium release, and reduced neuronal excitability) and causing impaired learning and memory (Gant et al., 2011).

## FAMILY OF AUTISM-RELATED DISEASES WITH DEFECTIVE NEURONAL CALCIUM SIGNALING

A number of diseases have long been recognized to be co-morbid with autism – seizures, migraine and BPD being the most prominent (Gargus, 2009, 2010). While there are many competing theories for such familial clustering, an important consideration is that these superficially distinct diseases share some fundamental components of vulnerability, likely arising from a shared subset of susceptibility-conferring loci. Recently this was explicitly shown to be true for the neuropsychiatric phenotypes of autism, ADHD, BPD, depression and schizophrenia. It was shown that these phenotypically distinct disorders, both childhood- and adult-onset, share common genetic alterations and hence, pathways (Psychiatric Genomics Consortium et al., 2013). In that study, voltage-gated calcium signaling was shown to be a joint common susceptibility factor for all various psychopathological conditions.

While these complex neurological diseases are caused overwhelmingly by heterogeneous genetic and environmental factors, the seizure and migraine etiological neuronal phenotypes are emerging from analysis of rare monogenic forms of these diseases. The monogenic seizure and migraine diseases are all caused by ion channel mutations and have a common channelopathy pathogenesis (Dodick and Gargus, 2008). The mutations all produce constitutionally hyper-excitable neurons that are susceptible to “arrhythmias” or periodic decompensations, much like the LQT heart or the MHS-periodically paralyzed muscle (Gargus, 2006, 2008, 2009). For example, the gene families (*FHM1/CACNA1A, FHM2/ATP1A2* and *FHM3/SCN1A*), the mutational lesions and the integrated pathophysiology underlying FHM3, are all strikingly homologous to those in epilepsy, LQT and MHS, and provide a robust platform for understanding the pathogenesis of the calcium channel autism syndrome, TS/LQT8. The *FHM3* and *FHM1* genes are paralogs of the sodium and calcium channel genes that contribute to MHS (*MHS2* and *MHS5*, respectively) and to LQT (*LQT3* and *LQT8*, respectively). The close relationship of the *FHM3* and autism alleles of *FHM3/SCN1A* has been discussed above.

The sum of the growing evidence supports the role of calcium signaling as one of the major participants in the pathogenesis of ASD, making it a promising therapeutic target. Furthermore, calcium signaling abnormalities have the potential to serve as functional biomarkers of the disease, a quantifiable objective measure independent of subjective behavioral assessments, and possibly useful as a cellular diagnostic in routine clinical care, complementing current complex neurobehavioral testing batteries.

## Conflict of Interest Statement

The authors declare that the research was conducted in the absence of any commercial or financial relationships that could be construed as a potential conflict of interest.
